# Involvement of Astrocytes in Mediating the Central Effects of Ghrelin

**DOI:** 10.3390/ijms18030536

**Published:** 2017-03-02

**Authors:** Laura M. Frago, Julie A. Chowen

**Affiliations:** 1 Department of Endocrinology, Hospital Infantil Universitario Niño Jesús, Instituto de Investigación la Princesa, CIBER de Obesidad Fisiopatología de la Obesidad y Nutrición (CIBEROBN), Instituto de Salud Carlos III, 28009 Madrid, Spain; laura.frago@uam.es; 2 Department of Pediatrics, Universidad Autónoma de Madrid, 28049 Madrid, Spain

**Keywords:** glia, neuroprotection, inflammation, metabolism

## Abstract

Although astrocytes are the most abundant cells in the mammalian brain, much remains to be learned about their molecular and functional features. Astrocytes express receptors for numerous hormones and metabolic factors, including the appetite-promoting hormone ghrelin. The metabolic effects of ghrelin are largely opposite to those of leptin, as it stimulates food intake and decreases energy expenditure. Ghrelin is also involved in glucose-sensing and glucose homeostasis. The widespread expression of the ghrelin receptor in the central nervous system suggests that this hormone is not only involved in metabolism, but also in other essential functions in the brain. In fact, ghrelin has been shown to promote cell survival and neuroprotection, with some studies exploring the use of ghrelin as a therapeutic agent against metabolic and neurodegenerative diseases. In this review, we highlight the possible role of glial cells as mediators of ghrelin’s actions within the brain.

## 1. Introduction

Ghrelin modulates systemic metabolism at least in part through activation of orexigenic neural circuits in the hypothalamus [1,2,3] and consistent with this, circulating levels of ghrelin rise promoting an increased sensation of hunger [4]. Ghrelin receptors are found on hypothalamic neurons that regulate food intake and satiety [5,6], but also on neurons in non-hypothalamic brain regions that contribute to eating behavior [7]. However, evidence supports a more complex role for this hormone in the brain and suggests that it participates in a variety of biological functions including modulation of reward systems [8,9] and learning and memory performance [10,11], as well as playing a protective role against degenerative diseases [12,13]. Although these effects are mainly mediated through ghrelin responsive neurons, glial cells such as astrocytes may also participate in these processes.

Astrocytes are involved in a wide range of functions that insure normal brain development and neural functioning [14] and are fundamental for processes of neuroprotection during both health and disease [15]. In recent years, the participation of astrocytes in both physiological and pathophysiological processes related to metabolic control has received increasing attention [16,17]. Indeed, these glial cells express receptors for numerous trophic factors and hormones, participating in and mediating the effects of these signals on the surrounding neurons. Astrocytes express the ghrelin receptor and respond to this hormone and its analogs [18,19,20], although there is little information to date regarding the physiological and/or pathophysiological function of these receptors in astrocytes. Here we have reviewed what is known to date regarding the response of astrocytes to ghrelin and how these glial cells might participate in mediating the effects of this hormone on appetite and neuroprotection.

## 2. Ghrelin and Its Receptor

The discovery of ghrelin, a gastrointestinal peptide, was reported in 1999 [21]. Kojima and colleagues showed that ghrelin was the endogenous ligand for the growth hormone secretagogue receptor (GHSR1a) and that it stimulated growth hormone (GH) release from the anterior pituitary gland. Indeed, ghrelin is the most potent peripherally produced endogenous inducer of the GH/insulin-like growth factor-1 (IGF-1) axis [22] and of food intake in mammals [23]. In 2000, Tschöep et al. demonstrated that ghrelin exerts actions in the brain to regulate food intake, body weight, adiposity, and glucose metabolism [24]. As stated above, ghrelin modulates systemic metabolism via activation of orexigenic neural circuits [1] and this is largely a direct response on these circuits as its receptor is highly expressed in the hypothalamic neurons that regulate food intake and satiety [25,26]. In addition, the GHSR1a is also expressed in extra-hypothalamic brain regions that modulate feeding behavior [27]. Other actions of this gut hormone that have been less studied include modulation of reward systems [28] together with learning and memory performance [10]. It also plays a protective role against neurodegenerative diseases [29,30,31]. Thus, ghrelin emerges as a hormone with a wide range of functions and possible therapeutic applications, although much is yet to be learned concerning its mechanisms of action.

In order to mediate many of its biological actions, the post-translational acylation of ghrelin by actions of the enzyme ghrelin *O*-acyltransferase [32,33] is necessary, with this chemical modification facilitating its binding to GHSR1a [34]. The majority of its physiological effects are mediated through this receptor, as ghrelin treatment does not stimulate GH release or an increase in food intake in GHSR1a-null mice [35]. Ghrelin binds to the third transmembrane domain of GHSR1a, a G protein-coupled receptor [36].

The mechanism by which circulating ghrelin can cross the blood–brain barrier (BBB) has been the subject of diverse studies and is still under debate, although it is clear that it does reach non-circumventricular areas as intravenous ghrelin administration was shown to induce Fos expression in these areas [37]. The octanoic group confers hydrophobicity to the N terminus of ghrelin and this facilitates its transport across the BBB where it is then able to perform its central actions [38]. In addition to the possible passive diffusion across the BBB, ghrelin is also reported to directly reach the hypothalamic arcuate nucleus through fenestrated capillaries [39]. In mice, a saturable carrier system has been described by which acyl-ghrelin crosses the BBB, but only in the brain-to-blood direction, whereas desacyl-ghrelin can be transported in both directions by a non-saturable mechanism. However human ghrelin, which differs from the mouse peptide in two amino acid residues, is reported to be bidirectionally transported across the BBB by a saturable system [10,40]. Thus, it appears that ghrelin transport across the BBB is finely regulated and involves complex mechanisms that appear to be different between species [41].

It was originally reported that the deacylated form of ghrelin does not signal through GHSR1a; however, desacyl-ghrelin can activate the GHSR1a receptor at supraphysiological doses [42,43] and intracerebroventricular (i.c.v.) injections of desacyl-ghrelin are reported to have physiological effects on metabolism via the GHSR1a [44]. Moreover, transgenic mice over-expressing desacyl-ghrelin are small, with the GH/IGF-1 axis being blunted [45]. This suggests that desacyl-ghrelin does indeed exert physiological functions, although more investigation is needed regarding the receptor involved and the underlying mechanisms.

There is also a truncated form of the ghrelin receptor, GHSR1b. This non-signaling receptor is suggested to exert a dominant negative role in the trafficking and signaling of GHRS1a [46]. Recent evidence indicates that GHSR1b determines the efficacy of ghrelin-induced GHSR1a-mediated signaling and facilitates the formation of oligomeric complexes of GHSR1a with other receptors, leading to changes in ghrelin-induced signaling [47].

## 3. Astrocytes

Astrocytes are the most abundant cell type in the central nervous system (CNS) and occupy more than 50% of total brain volume [48]. They are most often identified by the expression of the intermediate filament glial fibrillary acidic protein (GFAP), although not all astrocytes express this structural protein [49]. Indeed, these cells have diverse phenotypes depending on their location within the brain [50,51], exhibiting differential expression of receptors for a variety of molecules such as insulin [52], IGF-1 [53], dopamine, serotonin [54], estrogens [55,56], androgens [57], glutamate [58,59], gamma-aminobutyric acid (GABA) [60], ion channels [61], norepinephine [62], leptin and neuropeptide Y (NPY) [63]. They participate in the control of extracellular concentrations of ions and neurotransmitters [64], provide neurotrophic support [65], are involved in the formation, maintenance and functioning of synapses [66], modulate neuronal connectivity and synaptic efficacy [49], and contribute to the maintenance of the BBB [67]. After an injury, astrocytes undergo rapid changes that can either promote or prevent recovery depending on the type and extent of the damage [68,69]. The morphology and function of these glial cells depends on their activational state and is also rapidly modified in response to the activity of surrounding neurons [48,61] and by changes in Ca^2+^ signaling [70,71]. These changes can include modifications in their expression of glucose [72] and glutamate transporters [73,74,75] and in their coverage of neuronal membranes and synapses [76,77].

In response to inflammation or after brain damage, astroglia can be activated and proliferate with an increase in the expression of the structural proteins GFAP and vimentin [78,79,80]. This phenomenon is called astrogliosis and its main function is to protect the damaged area; however, glial scarring can occur in order to insulate or isolate the lesion, but this process also prevents the formation of new connections [81]. If reactive astroglia are removed after damage, there is an increase in the entry of inflammatory cells across the BBB with further extension of inflammatory process and increased neuronal loss [82]. Therefore, controversy remains concerning the beneficial implications of astrogliosis, at least under some circumstances [83].

Astrocytes are excitable cells but, unlike neurons, their excitability is not based on changes in membrane potential, but on changes in intracellular Ca^2+^ levels [84]. They release gliotransmitters (peptides, cytokines, and chemokines) to directly activate receptors on neighboring neurons and express transporters for the re-uptake of neurotransmitters [85]. Therefore, astrocytes are involved in modulating synaptic efficacy and maintaining ion and neurotransmitter homeostasis in the synaptic cleft and extracellular environment.

During primate evolution, the proportion of astrocytes to neurons has risen [86], suggesting that astrocytes play an important role in the increased complexity of the brain. The maturation of astrocytic processes and the establishment of spatial astrocyte domains in the brain coincide temporally with the formation of the first synapses [48]. Astrocytes are not only essential for synaptogenesis [48,87], but they also participate in synaptic remodeling, including that which occurs in neuroendocrine processes [88]. Hence, astrocytes regulate or modulate almost all neuronal functions, with some effects of hormones, trophic factors, nutrients, and other substances being mediated through these glial cells.

## 4. Ghrelin Receptors in Astrocytes

The ghrelin receptor is expressed in a wide variety of tissues and cell types throughout both the CNS and periphery [89,90]. In addition to its expression in neurons, GHSR1a has been shown to be expressed in astrocytes of the hypothalamic arcuate nucleus [19,20] and of the dentate gyrus in the hippocampus [18,91], although a thorough mapping of the expression of this receptor in astrocytes is yet to be performed.

The ghrelin receptor is also present in astrocytomas, with astrocytoma cell lines expressing higher levels of GHSR1a compared with primary cultures of normal astrocytes [92,93]. Ghrelin promotes cell motility in astrocytomas and, given that it is a positive regulator of the somatotropic axis, it could play a role in astrocytoma cell growth and function with an endogenous hormonal loop, critical in astrocytoma motility and invasiveness, possibly being involved [93]. On the contrary, desacyl-ghrelin, which does not bind GHSR1a at physiologic concentrations, does not exert this effect [94]. Moreover, ghrelin is unable to elicit any biological effect in GHSR1a-null astrocytoma cells, further suggesting that ghrelin induced astrocytoma cell motility is mediated by the GHSR1a receptor [93].

## 5. Ghrelin and Astrocytes in Metabolic Control

The involvement of astrocytes in metabolic control has been increasingly studied in recent years [48,95,96]. Astrocytes form part of the BBB and are located in close proximity to blood vessels, which facilitates their transport of ions and other substances, including nutrients and metabolic hormones, from the peripheral circulation into and within the brain. These nutrients not only serve as an energy source for cells of the brain but, in conjunction with hormones, inform nutrient/hormone sensing metabolic circuits as to the systemic metabolic status [97]. Glucose is the major energy source for cells of the CNS [98] and increased glucose transport is essential for maintaining homeostasis during synaptic activity [99], with this control depending on the communication between astrocytes and neurons [100]. Glucose transported into the brain by astrocytes can be used for their own survival, be stored as glycogen in order to regulate CNS glucose levels in situations of increased energy demand or be transported to neurons to be used as their energy source [101]. Hence, astrocytes are responsible for regulating the amount of glucose available in the extracellular space in the CNS [102]. The ability of astrocytes to transport glucose is modulated by metabolic hormones, including insulin [52], leptin [103], and ghrelin [19,20]. When astrocytes lack leptin receptors, as a result of experimental genetic manipulation, there is a reduction in the anorexigenic response to this hormone and there is a decreased response to fasting and to the effect of ghrelin on appetite [104]. The function of astrocytic insulin signaling is essential for hypothalamic glucose sensing and systemic glucose homeostasis [52,53]. Moreover, astrocytic insulin signaling is required for efficient glucose uptake into the brain in response to changes in systemic glucose availability. If insulin signaling in astrocytes is impaired, as occurs during diet-induced systemic insulin resistance, astrocyte morphology, mitochondrial function, and circuit connectivity is affected [52,53]. In addition, there is a reduction in glucose-induced activation of hypothalamic POMC neurons with the physiological response to changes in glucose availability being impaired [52]. Although central glucose transport or metabolism has not been analyzed in animals lacking GHSR1a specifically in astrocytes, acyl-ghrelin can modulate glucose uptake into hypothalamic astrocytes, at least in vitro [19]. The effect of ghrelin on glucose transport within the brain is further supported by the observation that the expression of glucose transporters in the hypothalamus is rapidly modified by icv administration of acyl-ghrelin [19]. In addition to modulating central energy availability, changing glucose transport by hypothalamic astrocytes in response to metabolic hormones such as ghrelin could also affect central glucose sensing [19,105], which would in turn modulate appetite and systemic glucose metabolism.

Under some circumstances, lactate can replace glucose as an alternative substrate in brain energy metabolism [106]. Lactate is produced and secreted by astrocytes with neurons then taking it up via monocarboxylate transporters [107,108] and converting it to pyruvate for oxidative production of ATP [106]. Glycogen stored in astrocytes can also be released as lactate to neurons [106,107,109]. Ghrelin stimulates the expression of glycogen phosphorylase, the rate limiting enzyme in glycogenolysis, in primary hypothalamic astrocyte cultures [19]. This could lead to increased hydrolysis of glycogen stores, producing an increase in lactate production and availability to neurons. Indeed, ghrelin also stimulated the expression of lactate dehydrogenase and of monocarboxylate transporter 4 (MCT4) in these cells [19]. It is enticing to postulate that ghrelin acts as a signal relaying systemic energy availability to astrocytes, which in turn modulate the energy substrates that neighboring neurons receive. If these neurons form part of the energy sensing metabolic circuits, ghrelin would modify the signals that they receive through astrocytes.

The opposite metabolic actions of ghrelin and leptin are due in part to their inverse effects on the release of hypothalamic neuropeptides, such as agouti-related protein (AgRP) and pro-opiomelanocortin (POMC) derived neuropeptides [110]. In addition, these two hormones also induce opposite changes in the synaptic organization of hypothalamic metabolic circuits [111] and astrocytes most likely participate in this reorganization [112]. In high fat diet (HFD)-induced obesity, astrocytic coverage of AgRP and POMC neurons is modified and this is inversely correlated with changes in synaptic inputs to the soma of these neurons [112,113,114]. Both weight gain and leptin administered icv modify hypothalamic astrocyte structural proteins and morphology, as well as the expression of synaptic proteins [115,116], with the knock out (KO) of leptin receptors specifically in GFAP-producing cells resulting in modifications in astroglial morphology and changes in the astrocyte coverage of hypothalamic metabolic neurons and their synaptic inputs [106]. Although icv administration of acyl-ghrelin was not found to modify hypothalamic GFAP levels or astrocyte morphology one hour after treatment, it did affect synaptic protein levels [19]. Moreover, vimentin levels and tanycyte projections were rapidly increased by icv acyl-ghrelin injection [19]. Tanycytes, specialized hypothalamic glial cells located in the median eminence transport peripheral hormones such as leptin [117] and ghrelin from the periphery to the arcuate nucleus. Alteration in ghrelin uptake by tanycytes is involved in the attenuated ghrelin transport observed after diet-induced obesity [118]. This, with the demonstration that both acyl- and desacyl-ghrelin modulate hypothalamic astrocytes in culture [19], indicates that the two isoforms of this hormone could possibly mediate effects on metabolic circuit organization through ghrelin.

Astrocytes also contribute to the control of food intake by directly modulating the actions of the orexigenic AgRP neurons located in the medial basal hypothalamus. Astrocytes release adenosine that inhibits ghrelin-evoked feeding by inactivating orexigenic AgRP neurons via adenosine a 1 (A1) receptor signaling [119]. Deletion of astrocytic leptin signaling also modified ghrelin and leptin-regulated feeding behaviors [104]. These studies indicate that astrocytes affect food intake by regulating the synaptic strength of metabolic control circuits and the activities of neurons controlling appetite and energy expenditure [119].

Astrocytes also modulate synaptic transmission and exert neuroprotective effects through their uptake of glutamate from the synaptic cleft [120] and ghrelin can affect their ability to do so. The glutamate transporters glutamate transporter 1 (GLT1) and glutamate aspartate transporter (GLAST) are highly expressed on astrocytes [121,122]. We have shown that ghrelin treatment icv rapidly stimulates GLT1 and GLAST levels, while only GLAST levels were found to increase in vitro [19]. Acyl-ghrelin rapidly induces the uptake of glutamate by hypothalamic astrocytes in vitro, with a reduction in uptake occurring with long-term exposure to this hormone [19]. Moreover, ghrelin stimulates glutamate release and elevates synaptic glutamate release which results in increased firing of AgRP neurons, response related increased with food intake in mice [123,124]. As mentioned above, ghrelin’s orexigenic properties are mediated by AgRP neurons with ghrelin increasing the activity of AgRP neurons, which in turn increases the release of GABA to inhibit POMC neurons [1]. However, when high fat and high sucrose diets are provided, AgRP neurons are dispensable for an appropriate feeding response [125], as well as possibly in glucodeprivation [126]. Andrews et al. [12] suggested a direct action of ghrelin on AgRP neurons through GHSR1a signaling and AMP-activated protein kinase (AMPK)-mediated alteration of fatty acid oxidation and regulation of mitochondrial uncoupling proteins [12]. The findings of Yang et al. [124] suggest that ghrelin acts at presynaptic receptors, increasing glutamate release and AgRP neurons are activated through ionotropic glutamate receptors [124]. This is not related to an increase in the number of excitatory synapses as shown previously [111]. The glutamate release induced by ghrelin also stimulates the uptake of glutamate by astrocytes in order to prevent excess excitability and excitotoxicity to neighboring cells [127,128,129,130].

Glutamate is metabolized to glutamine in astrocytes via glutamine synthase or it can be shuttled into the tricarboxylic acid cycle [99]. Glutamine released by astrocytes can be taken-up by neurons and reconverted into glutamate [131]. Ghrelin inhibits the expression of glutamine synthetase both in vivo and in vitro [19], which could indicate that glutamate metabolism is modified by this hormone. Indeed, the recycling of glutamate is coupled to glycolysis and glutamate can also be used as an energy source [106].

Ghrelin stimulates the production and release of GABA [132]. By means of the GABA-glutamine cycle that occurs at GABAergic synapses, neurons take up glutamine that is converted to glutamate that can then be metabolized to GABA by glutamate decarboxylase. Once released, GABA is taken-up by astrocytes through specific transporters and catabolized to succinate that enters the tricarboxylic acid cycle to generate glutamine [99]. Similarly fasting, possibly through stimulation of ghrelin secretion, increases hypothalamic GABA concentrations [133]. Hence, astrocytes take part in mediating ghrelin’s effects on hypothalamic synaptic transmission not only through modifications in neurotransmitter uptake, but also in their metabolism [19].

Studies indicate an important role for glial cells in pathological responses to excess weight gain [112]. Weight gain as a result of HFD intake is associated with activation of hypothalamic microglia and astrocytes, resulting in cytokine production and the activation of inflammatory signaling pathways in the hypothalamus [112,115,134] that can lead to central insulin/leptin resistance and metabolic disequilibrium [135]. Ghrelin exerts anti-inflammatory effects in various tissues [136,137,138] and in hypothalamic astrocyte cell cultures ghrelin decreased tumor necrosis factor-α (TNF-α) mRNA levels [20]. Thus, while ghrelin stimulates food intake and weight gain, it may also induce mechanisms of cell protection, at least in part through direct anti-inflammatory effects on astrocytes and this could help to delay systemic inflammatory responses and hypothalamic gliosis due to excess weight gain, which in turn would delay the onset of obesity-associated pathologies [20]. Obesity reduces plasma ghrelin concentrations which is a consequence of decreased secretion from gastric cells. In addition, the central responsiveness to ghrelin is reduced through reduction in the expression of GHSR1a on target neurons and through altered metabolic endocrine feedback in diet-induced obesity. This ghrelin resistance is suggested to possibly protect the system from establishing a higher body weight set-point during times of food availability and to maximize energy reserves which is one of the physiological functions of ghrelin: to defend body weight and glucose homeostasis [139]. However, it is unknown whether the response of astrocytes to ghrelin is modulated during obesity. Although o*b/ob* mice fed a HFD show gliosis in the arcuate nucleus, they remain ghrelin-sensitive suggesting that hypothalamic gliosis does not cause ghrelin resistance [140]. In contrast, in this same study [140] central leptin administration to *ob/ob* mice was found to induce ghrelin resistance. As leptin signaling in astrocytes modulates the response to ghrelin [105], astrocytes could be involved in this change in hormonal sensitivity.

The in vitro effects of ghrelin on astrocyte glucose and glutamate transport appear to be mediated mainly through GHSR1a, as acyl-ghrelin does not stimulate glucose transporter 2 (GLUT2) or GLAST levels in primary hypothalamic astrocyte cultures from *GHSR1a* KO mice [19]. Moreover, desacyl-ghrelin did not stimulate the expression levels of these two transporters in primary astrocyte cultures from normal rats, again suggesting that this effect is mediated though GHSR1a. In contrast, in rat astrocytes GFAP mRNA levels were increased by exposure to both acyl- and desacyl-ghrelin [19], suggesting that both isoforms of this hormone could affect the functioning of hypothalamic astrocytes. More studies are necessary to understand the full effects of these two isoforms on astrocytes and metabolic functioning.

## 6. Ghrelin and Astrocytes in Neuroprotection

Astrocyes are also critical to neuronal survival and repair [141]. After brain injury astrocytes become activated, resulting in morphological changes associated with the up-regulation of structural proteins such as GFAP and vimentin, but also changes in their release of cytokines, growth factors, and other signals to modulate neurons [142,143]. The growth, survival, and differentiation of neurons are dependent on autocrine and paracrine effects of neurotrophic factors and increased neurodegeneration occurs if astrocytes are not present. Neurotrophic factors secreted by astrocytes promote neuronal survival and morphological changes of these glial cells can minimize damage to neighboring neurons by the formation of a glial scar [144]. In situations of damage such as stroke, trauma, Alzheimer’s or Parkinson’s disease, reactive astrocytes clear glutamate and ions released from injured neurons and also clear metabolic byproducts in an attempt to maintain the local environment [145]. Several cytokines, including interleukin-1 (IL-1) and IL-6, have been implicated in the induction and modulation of reactive and pathological inflammatory responses [145]. However, in vitro data suggest that IL-1, IL-6, and TNF-α may be neuroprotective at lower doses and could support the production of neuroprotective mediators [146]. Thus, the primary objective of astrocyte activation in injury or in response to toxic substances is to protect the surrounding neurons.

Disruption of the BBB during traumatic brain injury is reported to be blunted by ghrelin treatment [147]. Neuronal degeneration and indices of brain tissue damage due to traumatic brain injury were decreased by ghrelin, which was related to the maintenance of BBB vascular permeability, protection against brain edema and reduction of astrocyte reactivity by this hormone [147]. It is proposed that ghrelin could act through an uncoupling protein 2 (UCP-2)-mediated mechanism to attenuate BBB disruption during injury [148]. In contrast, in response to stroke desacyl- but not acyl-ghrelin is reported to improve both functional and neurological outcomes after cerebral artery occlusion [149]. In this study, post-stroke treatment with desacyl-ghrelin decreased the infarct area and swelling and reduced BBB disruption [149]. These two isoforms could perform beneficial effects through different mechanisms and thus be more or less effective in activating protection processes depending on the type of injury, although this remains to be demonstrated.

Ghrelin acts as a survival factor for neurons through its inhibition of apoptotic pathways [150,151], having been shown to exert a protective role against a variety of stimuli including ischemia/reperfusion [152,153], alendronate [154], serum deprivation [155], doxorubicin [156], and TNF-α [157]. At least some of the neuroprotective effects of ghrelin are mediated through activation of GHSR1a and the subsequent stimulation of extracellular signal–regulated kinases (ERK1/2) and phosphatidylinositol-4,5-bisphosphate 3-kinase/protein kinase B or Akt (PI3K/Akt) pathways [151]. Systemic administration of the ghrelin mimetic growth hormone-releasing peptide-6 (GHRP-6) increases the central expression of proteins involved in cell survival and neuroprotection [158,159]. Treatment of adult male rats with GHRP-6 for one week significantly increased IGF-I mRNA levels in the hypothalamus, cerebellum, and hippocampus and activated the PI3K/Akt pathway and increased the levels of the anti-apoptotic protein B-cell lymphoma 2 (Bcl-2). Moreover, GHRP-6 reduced cerebellar cell death in aged rats via the stimulation of IGF-I production and inhibition of caspases 9 and 3 [160]. Other studies report that ghrelin exerts its neuroprotective effects through stimulation of the protein kinase A and C pathways [151]. GHRP-6 is capable of preventing glutamate-induced neuronal death in both the hypothalamus and cerebellum [129] and also in the hypothalamic neuronal cell line RCA-6 [127]. Ghrelin has a neuroprotective role in hippocampal neurons against KA-induced excitotoxicity [161] via activation of the PI3K/AKT pathway and inhibition of the mitochondrial apoptotic pathway. In studies in adult rats treated with GHRP-6, no change in GFAP or vimentin levels were found, but activation of the PI3K/Akt pathway was observed and this was associated with increased markers of proliferation. The astrocytoma cell line C6 also responds to GHRP-6 by up-regulating GHSR1a levels, increasing the activation of the PI3K/Akt pathway and increasing proliferation [93]. This effect is mediated by GHSR1a as d-Lys3-GHRP-6, an antagonist of GHSR1a, reduced the GHRP-6 stimulated increase in cell number [18], suggesting that ghrelin could possibly stimulate the proliferation of astrocytes through GHSR1a.

Chronic administration of acyl-ghrelin was shown to be protective in a mouse model of Parkinson’s disease [12] and mice lacking both desacyl- and acyl-ghrelin (*ghrelin* KO mice) have enhanced neurodegeneration. In humans, Parkinson’s disease is associated with increased body mass, adiposity, and diabetes [162,163,164]; indeed, obesity is considered a risk factor for increased neurodegeration [165,166,167,168]. Ghrelin levels are inversely related to body mass; ghrelin levels are lower in obese subjects and are increased during caloric restriction [169] and studies show that this could affect neuroprotection. For example, hepatocytes and neurons treated in vitro with serum from caloric restricted rats had a reduction in the production of reactive oxygen species (ROS) [170]. When ghrelin levels are increased, 1-methyl-4-phenyl-1,2,5,6-tetrahydropyridine (MPTP)-induced neurotoxicity is attenuated [171,172]. Indeed, this hormone has been shown to preserve mitochondrial integrity and metabolism during oxygen–glucose deprivation [173] and to attenuate MPTP neurotoxicity in dopaminergic (DA) neurons [174] by improving mitochondrial function [175]. The protection induced by ghrelin against MPTP-induced neurotoxicity in nigral dopaminergic neurons in vivo involves a GHSR1a mediated anti-apoptotic effect [12]. Ghrelin could promote at least part of its neuroprotective actions by means of UCP2, as it has a role in buffering ROS production, enhancing mitochondrial biogenesis, and in respiration [176,177].

Situations that are associated with inflammation result in the activation of microglia and astrocytes, increasing the release of pro-inflammatory cytokines centrally, a process common to all neurodegerative diseases [178,179], ischemic brain injury [180], and traumatic brain injury [181]. To alleviate the symptoms of these diseases or injuries, anti-inflammatory agents are used. In both in vivo and in vitro studies, ghrelin has been shown to act as an anti-inflammatory mediator in response to different inflammatory situations and in different organs, including in brain injury and pain [182,183,184]. Ghrelin can inhibit microglial activation [129] and oligodendrocyte cell death that are co-cultured with lipopolysaccharide (LPS)-stimulated BV2 cells, a microglial cell line [185]. Moreover, *ghrelin* KO mice demonstrate an increased activation of microglia and astrocytes following cerebral ischaemia compared to wild type (WT) mice [186]. The role of ghrelin signaling in the neuroprotective effects of calorie restriction in Parkinson’s disease was explored with MPTP treatment in *ghrelin* KO mice. MPTP treatment increased GFAP positive cells in the substantia nigra in both WT and *ghrelin* KO mice fed ad libitum. Calorie restriction reduced GFAP levels in both MPTP-treated WT and *ghrelin* KO mice indicating that the caloric restriction-induced reduction in GFAP expression in the substantia nigra is not mediated by ghrelin [187]. Increased activation and accumulation of microglia and astroglia, which participates in the hippocampal neurodegeneration that follows kainate-induced excitoxic injury, is inhibited by ghrelin treatment [188]. Ghrelin suppress the kainic acid-induced increase in GFAP, as well as in TNF-α, IL-1β and cyclooxygenase (COX)-2 immunoreactivity, and matrix metalloproteinase-3 expression in the hippocampus. Ghrelin also attenuates the synthesis of TNF-α following traumatic brain injury [189].

**Figure 1:**
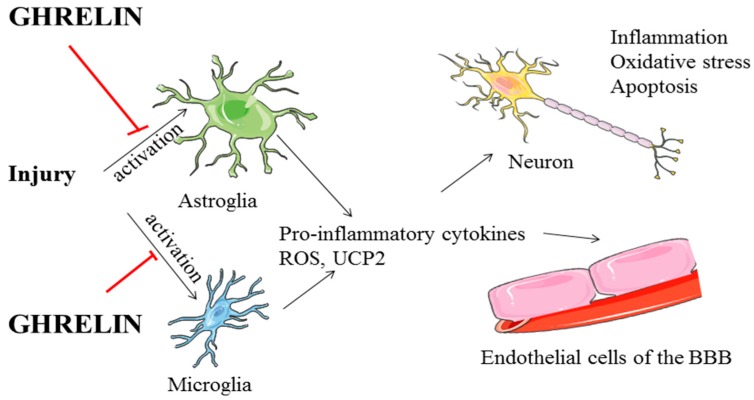
A schematic drawing of the regulation of inflammation and damage exerted by ghrelin in the brain. Ghrelin would prevent the activation of astroglia and microglia avoiding the excess release of pro-inflammatory factors that would affect neurons and endothelial cells in neurodegenerative or injury processes. BBB: blood–brain barrier, ROS: reactive oxygen species, UCP2. Uncoupling protein 2. Red bars indicate inhibition.

In summary, during infections, injuries, and seizures the first cells to produce cytokines in the brain are microglia and astrocytes, with these glial cells being the main sources of locally produced proinflammatory molecules (Figure 1). This inflammatory process then affects neurons and endothelial cells of the BBB [190], potentially potentiating the damage. Ghrelin exerts its neuroprotective effects, at least in part, through the inhibition of glial cell activation and production of pro-inflammatory neurotoxic mediators derived from activated glia, with many of these effects most likely being mediated through GHSR1a.

## 7. Conclusions

Less than 20 years have passed since the discovery of ghrelin was first reported and during this time diverse functions have been attributed to this hormone. However, many fundamental questions still remain. For example, it is clear that acyl-ghrelin has non-GHRS1a-mediated physiological effects and that desacyl-ghrelin has more physiological effects than originally thought; however, the receptors/mechanisms that mediate these actions remain to a large extent elusive. In addition, how this hormone influences astrocytes is only beginning to be investigated with much yet to be learned.

**Figure 2:**
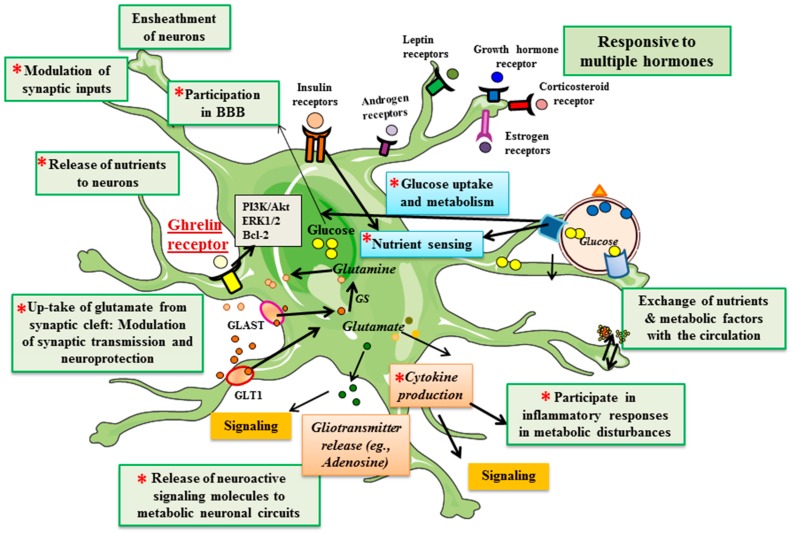
Schematic representation of the various functions attributed to astrocytes. Those marked with the red asterisks have been shown to be or are suggested to be modulated by ghrelin. BBB: blood–brain barrier, GS: glutamine synthetase, GLAST: glutamate aspartate transporter, GLT1: glutamate transporter 1.

The list of functions performed by astroglial cells continues to grow. It is now clear that hypothalamic astrocytes participate in the control of energy homeostasis, with metabolic hormones and nutrients mediating at least part of their effects on metabolic circuits through these glial cells. Ghrelin modulates astrocytic function including glucose transport, glucose metabolism, cytokine production, and trophic factor production (Figure 2). However, a lot is still to be learned regarding the full range of effects that this hormone exerts on these glial cells and how this could participate in mediating the orexigenic effects of ghrelin on the hypothalamus. Moreover, it is yet to be determined if ghrelin affects glucose transport and metabolism in astrocytes throughout the brain or if this action is specific to the hypothalamus. If specific to this brain region, it could indicate a more direct implication in systemic metabolic control compared to a more broad effect on local glucose transport/consumption.

Astrocytes also play a fundamental role in neuroprotection and ghrelin can prevent the activation of these glial cells, at least in some circumstances. As hypothalamic inflammation/gliosis is thought to perpetuate and augment food intake and weight gain, the rise in ghrelin before appetite increases could be a physiological protective mechanism against the possible harmful effects of increased food intake and metabolism. This hypothesis deserves further investigation. Indeed, more investigation is needed in order to understand astrocyte functions throughout the brain. It is clear that they are a heterogeneous population of cells, although we continue to lack appropriate tools to clearly identify and characterize these subpopulations. Even within the same anatomical area these cells can differ in something as elementary as the expression of GFAP.

With the increasing attention that astrocytes have received in recent years, including the attempt to understand their response to metabolic hormones, advances have made in our understanding of their importance in neuroendocrine function. This increased attention on astrocytes, in conjunction with the constant technological advances, will surely result in exciting new discoveries in the near future.
